# Tumour-suppression function of KLF12 through regulation of anoikis

**DOI:** 10.1038/onc.2015.394

**Published:** 2015-10-12

**Authors:** N Godin-Heymann, S Brabetz, M M Murillo, M Saponaro, C R Santos, A Lobley, P East, P Chakravarty, N Matthews, G Kelly, S Jordan, E Castellano, J Downward

**Affiliations:** 1 Signal Transduction, Cancer Research UK London Research Institute, London, UK; 2 The Institute of Cancer Research, London, UK; 3 Mechanisms of Gene Transcription Laboratory, Cancer Research UK London Research Institute, Clare Hall Laboratories, Hertfordshire, UK; 4 Translational Cancer Therapeutics, Cancer Research UK London Research Institute, London, UK; 5 Bioinformatics and Biostatistics Laboratories, Cancer Research UK London Research Institute, London, UK; 6 Advanced Sequencing Facility, Cancer Research UK London Research Institute, London, UK

## Abstract

Suppression of detachment-induced cell death, known as anoikis, is an essential step for cancer metastasis to occur. We report here that expression of KLF12, a member of the Kruppel-like family of transcription factors, is downregulated in lung cancer cell lines that have been selected to grow in the absence of cell adhesion. Knockdown of KLF12 in parental cells results in decreased apoptosis following cell detachment from matrix. KLF12 regulates anoikis by promoting the cell cycle transition through S phase and therefore cell proliferation. Reduced expression levels of KLF12 results in increased ability of lung cancer cells to form tumours *in vivo* and is associated with poorer survival in lung cancer patients. We therefore identify KLF12 as a novel metastasis-suppressor gene whose loss of function is associated with anoikis resistance through control of the cell cycle.

## Introduction

Metastasis is a multi-step process involving tumour cells leaving their site of origin, spreading via blood or lymph vessels and forming new tumours at distant sites. Detachment-induced cell death is an early step in preventing metastasis. When an untransformed cell detaches from its surrounding matrix or loses interaction with its neighbouring cells, it undergoes a particular type of apoptosis known as anoikis.^1^ Tumour cells that have the capacity to form metastasis have developed mechanisms to block anoikis. Improving our understanding of anoikis resistance could lead to the identification of novel potential therapeutic targets.

The effect of the extracellular matrix (ECM) on cells is mainly mediated by integrins, a family of transmembrane receptors that bind to the ECM and transduce intracellular signalling pathways. Upon integrin-mediated adhesion, both FAK and SRC are activated and they in turn activate various pathways such as phosphatidylinositol 3′-kinase/AKT, RAS/RAF/MEK/extracellular signal–regulated kinase (ERK) and nuclear factor-κB, resulting in overall survival signals.^1, 2^ However, when the integrin signal is lost due to cell detachment, these survival pathways are no longer dominant and anoikis occurs. Metastatic cells have developed various mechanisms to overcome anoikis, including epithelial-to-mesenchymal transition, changes in integrin repertoire, integrin internalization, constitutive activation of pro-survival signals such as autocrine secretion of growth factors or receptor tyrosine kinase overexpression, oxidative stress, autophagy or entosis.^3, 4^

Apoptosis has been closely linked to the cell cycle as various proteins are master regulators of both processes. Most prominently, p53 not only regulates the G1 and G2/M phases of the cell cycle, the spindle checkpoint and centrosome duplication but is also a major trigger of apoptosis.^5^ Other regulators that both stimulate proliferation as well as inducing apoptosis include the Myc-Max transcription factor complex and E2F1.^5^ Cyclin-dependent kinase 1 (CDK1), Cyclin A/CDK2 and cylcin D are required for cell cycle progression and have been shown to be essential for inducing apoptosis in some systems.^5, 6, 7, 8^

More specifically, anoikis sensitivity is associated with cell cycle regulation. Invasive and motile mesenchymal cells can survive without ECM interactions and become arrested at the G1 stage of cell cycle.^9, 10^ Similarly, a population of keratinocytes that survive in suspension undergo G0/G1 arrest,^11^ breast epithelial cells overexpressing galectin-3 are resistant to anoikis in a manner dependent on their arrest in G1^12^ and mammary epithelial cells can acquire anoikis resistance following a complete withdrawal from the cell cycle.^13^ It has been proposed that late G1 arrest is an anoikis-insensitive point.^12^ Brugge and colleagues have shown that MCF10A cells arrested in G1 or early S phase provide resistance to anoikis by suppressing BIM expression in a posttranscriptionally dependent manner.^14^

The Kruppel-like family (KLF) of transcription factors regulate multiple processes, such as proliferation, differentiation, migration and pluripotency.^15^ Moreover, KLF17 has been shown to be a repressor of metastasis.^16^ KLFs can activate and repress genes that participate in cell cycle regulation. They can be deregulated in multiple cancers either by loss of heterozygosity, somatic mutations or transcriptional silencing by promoter methylation.^17^

KLF12 was initially identified as a repressor for the transcription factor AP-2α.^18^ Amplification of the chromosomal region 13q21-13q22 harbouring KLF12 occurs in salivary gland tumours^19^ and poorly differentiated gastric cancers have increased expression of KLF12 that correlate with tumour size,^20^ suggesting a possible oncogenic role. However, the same chromosomal region houses a putative susceptibility gene in breast, prostrate and pancreatic cancer and is the site of somatic deletions in different malignant tumours.^21, 22, 23, 24^

In order to identify novel regulators of anoikis, we generated three human lung cancer sublines that were able to survive and proliferate in suspension. Microarray analysis of these suspension sublines relative to their parental cell lines showed that all three had decreased expression of KLF12. Functional analysis showed that knockdown of KLF12 in the parental cells could supress anoikis by slowing down S phase. Tail-vein assays confirmed the role of KLF12 as a suppressor of metastatic colony formation and higher expression levels of KLF12 correlate with increased survival of lung cancer patients in clinical data sets.

## Results

### Characterizing anoikis-resistant cancer sublines

**Figure 1:**
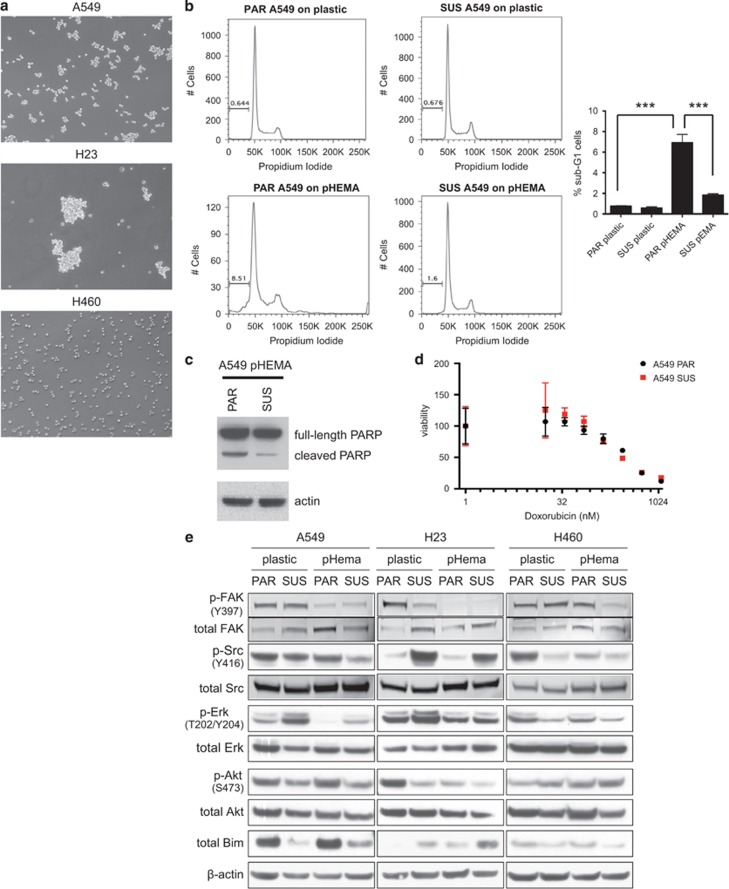
Characterization of the suspension-derived sublines. (**a**) Light microscopy images (× 10) of A549, H23 and H460 SUS cell lines growing in suspension (on polyHEMA) for at least 48 h. (**b**) A549 SUS cells have reduced sub-G1 population relative to A549 PAR cells when grown on polyHEMA. DNA content assessed by fluorescence-activated cell sorting. The percentage of the sub-G1 population is labelled (left). Histogram showing an average sub-G1 apoptotic population (*n*=3) (right). Following a one-way analysis of variance, the *P*-values were determined to be 0.00003 comparing PAR plastic and PAR pHEMA and 0.000124 comparing PAR pHEMA and SUS pHEMA, *n*=3. (**c**) Apoptosis induction assessed by poly ADP-ribose polymerase cleavage in A549 PAR compared with attenuated response in SUS cells grown on polyHEMA. (**d**) A549 PAR and SUS cell lines were treated in triplicates with serial dilutions of doxorubicin in adherent conditions. (**e**) Cell signalling profiling of parental and suspension A549, H23 and H460. Cells were cultured for 48 h on plastic or on polyHEMA. Cell extracts were subjected to immunoblotting with p-FAK(Y397), p-Src (Y416), p-Erk (T202/Y204), p-Akt (S473), Bim, total FAK, Src, Erk, Akt and β-actin antibodies.

In order to further our understanding of anoikis, we generated three anoikis-resistant human lung cancer sublines derived from the well-characterized NCI-60 adherent cell lines A549, H23 and H460. These sublines were selected by initially growing the cells to overconfluency, collecting the floating cells and growing these in adherent conditions. This cycle was repeated 3–4 times, after which the floating cells were selected for growth in suspension on polyHEMA-coated dishes to prevent the cells from adhering. Dead and dying cells were eliminated by growing the cells on plastic every few passages. The three suspension-derived cell lines differed in their macroscopic phenotype when grown on polyHEMA-coated plates: H460 grew as single cells or pairs of cells and A549 formed small clumps, whereas H23 grew in large clumps (Figure 1a).

Assessment of caspase activity of all three suspension-derived (SUS) cells grown on polyHEMA was significantly lower compared with parental (PAR) cells grown on polyHEMA, showing an increased resistance to anoikis under these conditions (Supplementary Figure S1A). As the A549 cell line had an intermediate phenotype when grown in suspension, further characterization was performed on this cell line. Cell cycle profiling confirmed that A549 SUS cells were more resistant to anoikis than the A549 PAR with a sub-G1 population fivefold lower (Figure 1b). This was further validated by lower levels of cleaved poly ADP-ribose polymerase in A549 SUS relative to A549 PAR when grown in suspension (Figure 1c). To assess whether this increased resistance is specific to anoikis or a general resistance to apoptosis, A549 PAR and SUS cells were treated with increasing doses of doxorubicin (Figure 1d). Both cell lines had a similar sensitivity to the drug, confirming that the SUS cells were selected to be specifically resistant to anoikis rather than more broadly to apoptosis.

As it has been well documented that overcoming metabolic stress can have an important role in anoikis resistance,^25^ we enquired whether there are any metabolic differences between the parental and suspension cell sublines that could explain how these sublines have acquired resistance. When comparing the basal oxygen consumption rate (OCR) of PAR and SUS cells, no differences could be detected for A549 and H460 cells, whereas a drastic reduction in basal OCR was observed in H23 SUS cells (Supplementary Figure S1B). We split the basal OCR into mitochondrial ATP production, mitochondrial proton leak and residual oxygen consumption by a sequential treatment with oligomycin, antimycin A and rotenone. Thus the loss of basal OCR in H23 SUS could be pinpointed to a drop in mitochondrial ATP production and proton leak while the residual oxygen consumption remained unchanged (Supplementary Figure S1C). No differences, however, were observed in the basal extracellular acidification rate (data not shown). As previous research has already linked a reduced respiratory chain activity to anoikis resistance, this observation might have a central role in the anoikis resistance of H23 SUS cells.^26^

In order to further characterize the PAR and SUS cell lines, we tested the activity of core adhesion and anoikis pathways in all three cell models growing on plastic and polyHEMA (Figure 1e). This analysis showed a heterogeneous result. All three cell lines adapted differently to the anchorage-independent growth condition. The A549 SUS cells show elevated p-Erk levels and lower levels of the proapoptotic Bim protein. This mechanism of anoikis resistance has already been characterized, because activated Erk1/2 phosphorylates BIM and this phosphorylation targets Bim for ubiquitination and proteasomal degradation.^27^ As well as their previously observed specific metabolic phenotype, H23 SUS cells are characterized by a high p-Src level that also has been correlated to anoikis resistance in lung adenocarcinoma cells.^28^ H460 SUS cells show slightly higher p-Akt levels, a commonly activated pathway in anoikis resistance.^2^ All three cell lines have reduced levels of phospho-FAK when grown on polyHEMA relative to when they are grown on plastic. As they are not attached to any ECM when grown on polyHEMA, one would expect reduced signalling from FAK in those conditions.

### KLF12 is downregulated in all three SUS-derived cell lines

Although differences have been observed in metabolism and signalling in some of the SUS-derived cell lines relative to their PAR cells, no single underlying mechanism of anoikis resistance was identified across all three cell lines. In order to uncover such a mechanism of resistance, a gene expression microarray analysis was performed on A549, H23 and H460 PAR cells grown on plastic and SUS cells grown either on plastic or polyHEMA.

**Figure 2:**
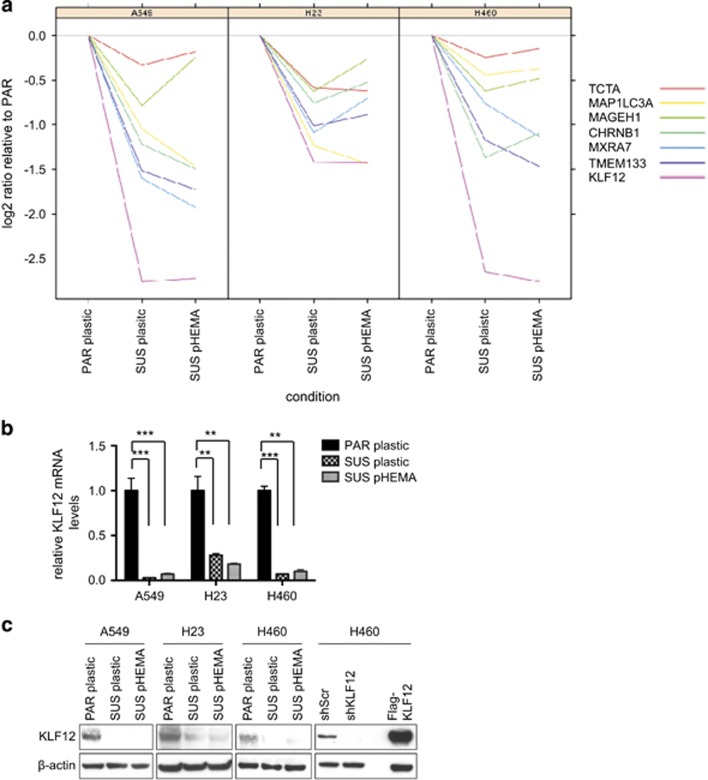
KLF12 mRNA levels are decreased in suspension-derived cell lines relative to parental cell lines. (**a**) A Nimblegen gene expression microarray analysis was performed on A549, H23 and H460 PAR cells in adherent conditions (PAR plastic) and their suspension derivatives grown in either adherent (SUS plastic) or suspension conditions (SUS polyHEMA). (**b**) Validating KLF12 reduced mRNA levels in triplicate in SUS-derived cell lines relative to PAR cells by Q-PCR. The *P*-values were determined using a Hochberg analysis and are 0.0005 and 0.00004 for A549 comparing PAR to PL and PAR to PH, respectively; 0.002 and 0.002 for H23 comparing PAR to PL and PAR to PH, respectively; and 0.0002 and 0.002 for H460 comparing PAR to PL and PAR to PH, respectively. (**c**) Western blotting showing KLF12 protein levels are decreased in SUS-derived cell lines relative to PAR cells.

The gene with the largest differential expression in all three cell lines was *KLF12* (Figure 2a), with at least twofold difference in expression in each of the cell lines between the PAR cells and the SUS cell lines grown on plastic.

Quantitative PCR (Q-PCR) analysis confirmed that KLF12 mRNA levels were decreased in the SUS cells relative to the PAR cells between 3- and 30-fold depending on the cell lines, irrespective of whether they were grown in adherent conditions on plastic or in suspension on polyHEMA (Figure 2b). Thus the selection for decreased levels of KLF12 mRNA is long term, as growth of the SUS cells on plastic for several passages did not revert KLF12 levels to those of the PAR cells. To confirm that the differential KLF12 mRNA levels were reflected at the protein level, an immunoblot was performed in all the cell lines (Figure 2c). As the specificity of the antibody had previously not been validated, it was confirmed by short hairpin RNA (shRNA)-mediated knockdown and also overexpression of a Flag-KLF12 protein (Figure 2c).

### KLF12 regulates anoikis

**Figure 3:**
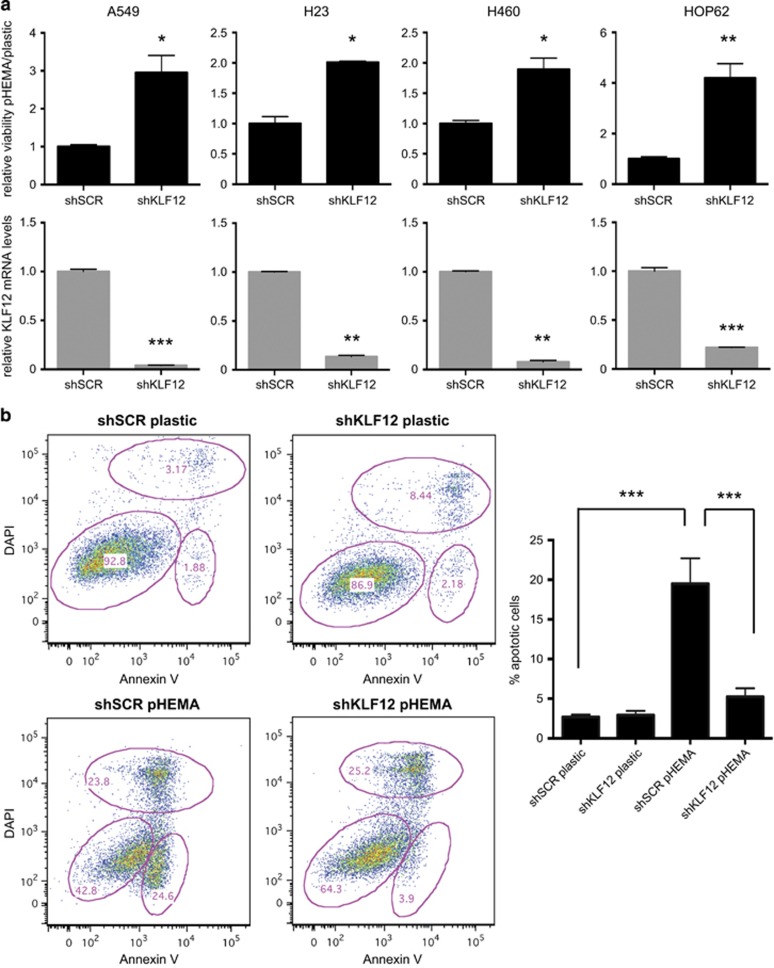
KLF12 promotes anoikis. (**a**) KLF12 knockdown results in decreased viability in suspension. A549, H23, H460 and HOP62 PAR cells infected with shRNA against KLF12 or scramble control were grown in plastic or polyHEMA and their viability was assessed (upper panels). The statistical significance was determined by Hochberg analysis (*n*=3) and the *P*-values were 0.02, 0.02, 0.02 and 0.008 for A549, H23, H460 and HOP62, respectively. KLF12 knockdown efficiently was determined in triplicate by Q-PCR (lower panels), and the statistical significance was determined by Hochberg analysis. The *P*-values were 0.0005, 0.005, 0.005 and 0.0004 for A549, H23, H460 and HOP62, respectively. (**b**) KLF12 knockdown leads to decreased apoptosis in suspension. A549 PAR cells infected with shRNA against KLF12 or scramble control were grown in polyHEMA or plastic and the Annexin-positive apoptotic population was quantified by fluorescence-activated cell sorting analysis (left). Histogram showing the Annexin-positive population as an average of four independent experiments (right). Following a two-way analysis of variance, the *P*-values were determined to be 0.00008 looking at the KLF12 effect within polyHEMA and 0.0005 looking at the polyHEMA effect within shSCR (*n*=4).

To assess whether KLF12 expression levels correlates with a functional effect on anoikis, an shRNA hairpin was used to knock down its expression in the three lung cancer cell lines used to generate anoikis-resistant cell lines (A549, H23, H460) as well as the additional lung cancer cell line HOP62 that had higher levels of *KLF12* expression. KLF12 knockdown was efficient in all four parental cell lines (Figure 3a, bottom panels) and this correlated with a twofold to fourfold increase in viability of the cells on polyHEMA relative to a scrambled control (Figure 3a, top panels). Moreover, the apoptotic population of A549 PAR cells grown on polyHEMA was significantly reduced in cells in which KLF12 was knocked down by shRNA (Figure 3b). A similar effect was seen with alternative knockdown approaches, using either a pool of small interfering RNA (siRNA) oligos against KLF12 (Supplementary Figure S2A) or an siRNA oligo against the 3′ untranslated region of KLF12 (Supplementary Figure S2B), indicating that the observed phenotype was not due to an off-target effect. In all cases, the apoptotic population was significantly reduced upon KLF12 knockdown when the cells were grown on polyHEMA, confirming a role of KLF12 in anoikis regulation.

### KLF12 has a role in cell cycle regulation

KLF12 is a member of the KLF of transcription factor and has previously been described as a repressor of the transcription factor AP-2α.^18^ AP-2α mRNA was not differentially expressed in the PAR compared with the SUS cell lines across all three cell lines either in the microarray data or by Q-PCR analysis (Supplementary Figure S3A and data not shown), suggesting an alternative mechanism for the effect of KLF12 on anoikis.

In order to provide an unbiased analysis of genes regulated by KLF12, the *KLF12* gene was silenced in PAR A549 by either siRNA or shRNA, and an RNAseq was performed in triplicate. Interestingly, there were significantly more genes differentially expressed following siRNA-mediated knockdown of KLF12 relative to shRNA-mediated knockdown, namely 5039 versus 2142. This could be due to the siRNA pool having increased off-target effects relative to the shRNA hairpin or to a more efficient level of KLF12 silencing by siRNA. A selection of genes that were shown to be upregulated or downregulated in KLF12 siRNA by RNAseq were validated by Q-PCR giving confidence in the results (Supplementary Figures S3B and C).

As integrin signalling has a key role in anoikis regulation, we wanted to establish whether KLF12 was involved in that pathway. Indeed, we identified a significant integrin signature using DAVID^29^ within our differentially expressed genes following KLF12 knockdown by siRNA; however, it was not statistically significant following KLF12 knockdown by shRNA (Supplementary Figure S3D).

**Figure 4:**
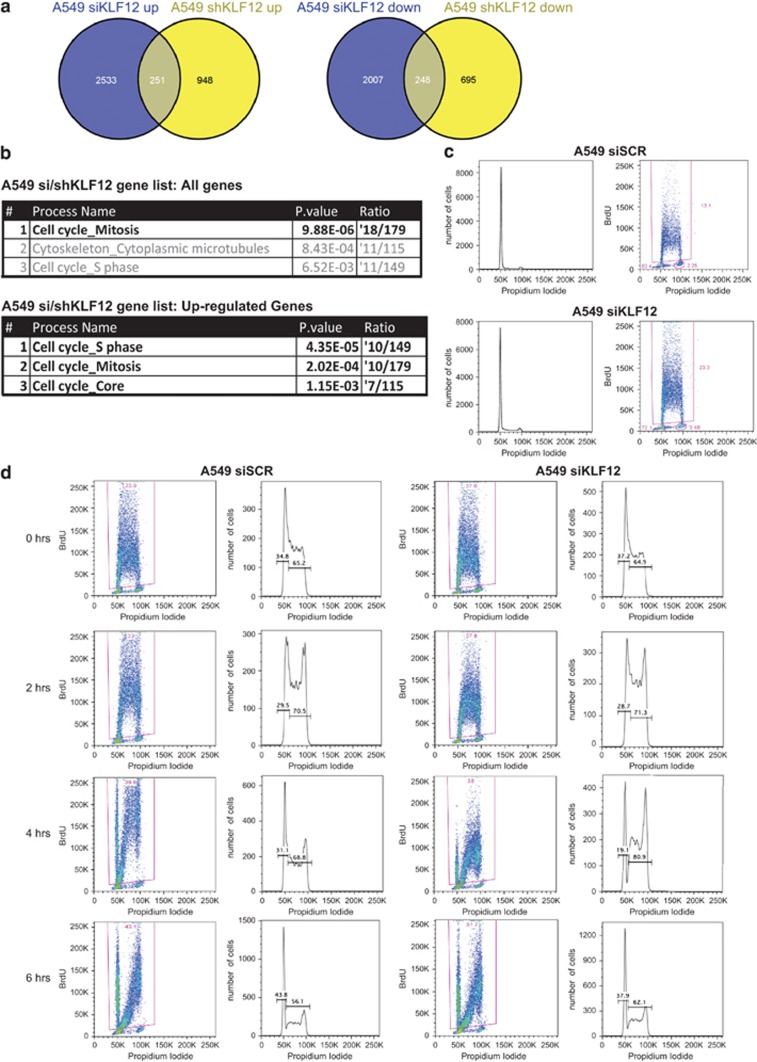
KLF12 regulates the cell cycle. (**a**) Venn diagram representation of RNA seq profile of genes upregulated (left) or downregulated (right) upon knockdown by shRNA against KLF12 or by siRNA against KLF12 in A549 cells. (**b**) KLF12 regulates genes involved in cell cycle in A549 cells. Gene Ontology (GO) process enrichment of all genes differentially expressed in siKFL12 and shKLF12 groups that overlap (top) and those that overlap from upregulated groups following siKLF12 and shKLF12 knockdown (bottom). (**c**) KLF12 knockdown leads to decreased S phase population in A549 cells. Left: Cell cycle profiling of A549 fluorescence-activated cell sorting. Right: DNA content by propidium iodide staining and BrdU content by anti-BrdU staining. The numbers of BrdU-positive cells representing S phase were determined. (**d**) KLF12 knockdown results in a lag of exiting S phase in A549 cells. Cells were transfected with a pool of siRNA against KLF12 or scramble control. Forty-eight hours after siRNA transfection, BrdU was pulsed for 15 min and then chased with BrdU-free medium, and cells were fixed at the indicated time points. For each time point and sample, BrdU-positive cells cycling through S phase are gated (left), and the DNA content of those cells are depicted (right). The percentage of cells in early S phase and mid-late S phase is shown.

To increase the confidence that we selected targets of KLF12, we analysed the genes that overlapped in the siRNA- and shRNA-mediated knockdown RNAseq in the same direction (Figure 4a). Gene ontology analysis using Metacore's Pathway tool identified cell cycle as the most significant process in all of the overlapping genes following RNA interference, as well as in those genes that were upregulated after siRNA and shRNA knockdown of KLF12 (Figure 4b). Q-PCR analysis confirmed the differential expression of these cell cycle genes following KLF12 knockdown (Supplementary Figure S4A). Indeed, knockdown of KLF12 in A549 increased the percentage of bromodeoxyuridine (BrdU)-positive cells undergoing S phase, thereby confirming a role of KLF12 in cell cycle regulation (Figure 4c).

An increase of BrdU-positive population could be due to either an increase in cell cycle, whereby more cells are cycling and therefore in S phase, or a slowing down/blockage of cell cycle in S phase, resulting in an accumulation of cells in mid–late S phase. To differentiate between these two opposing possibilities, a BrdU pulse chase was performed and the length of the cell cycle was determined in cells with KLF12 knockdown relative to scrambled control. Whereas the initial steps of cell cycle showed no difference between siKLF12 and scramble samples, a delay occurred 4 h after the BrdU pulse, with 69% BrdU-positive cells in mid–late S phase in scramble samples compared with 81% of cells that had decreased levels of KLF12 (Figure 4d). This delay in S-phase exit was short-lived as 6 h after the pulse the two samples showed similar BrdU profiles. To investigate whether this effect was specific to A549 or more general, a pulse chase was also performed in H460 cells with KLF12 knocked down or scramble control. As with A549, KLF12 silencing in H460 showed a delay in mid–late S phase (Supplementary Figure S4B). Thus KLF12 knockdown results in a small but consistent delay to exit S phase.

**Figure 5:**
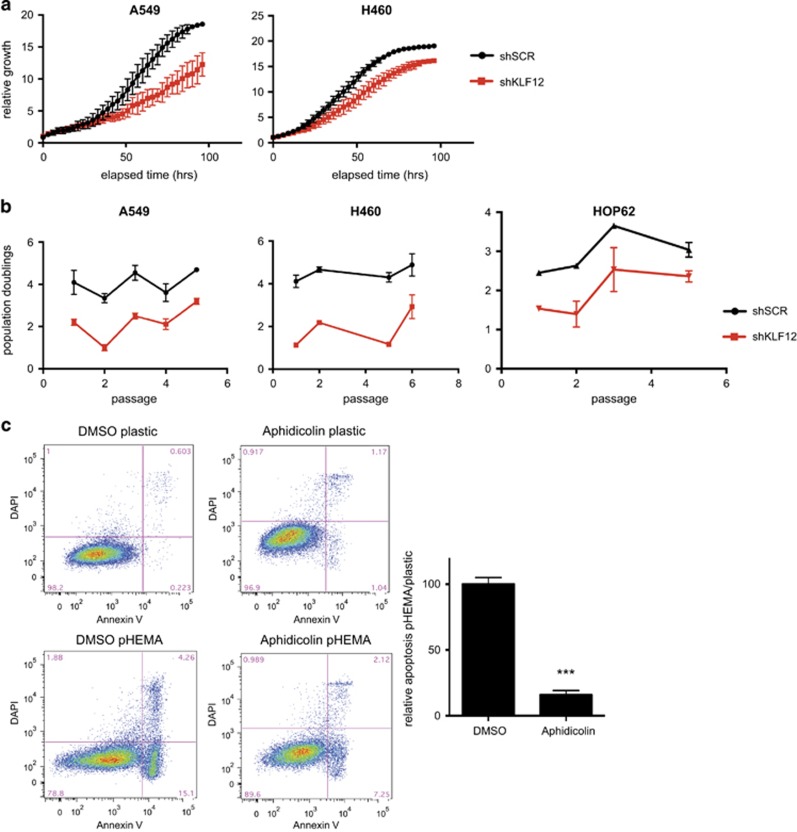
KLF12 regulates proliferation. (**a**) Reduced cell growth following KLF12 knockdown. Cell growth of A549 and H460 infected with shRNA against KLF12, as measured in real time in triplicate on an IncuCyte FLR (Essen Instruments) every 3 min. (**b**) Reduced proliferation in cells with decreased KLF12 levels. A549, H460 and HOP62 cells were infected with shRNA against KLF12 or scramble control (*n*=2 per condition). Cells were counted every 3–4 days by haemocytometer and re-seeded at a determined confluency. *P*-values for A549, H460 and HOP62 were 1.88 × 10^−15^, 1.10 × 10^−18^ and 4.15 × 10^−7^, respectively. The *P*-values were calculated by a three-way analysis of variance test using passage number and KLF12 status, nested within cell line, as the three covariates. Walt test was used against the interaction between cell line and KLF12 status. (**c**) Aphidicolin treatment reduces anoikis in A549. A549 cells were treated with 0.1 ug/ml Aphidicolin for 24 h after which the cells were grown either on plastic or polyHEMA for a further 48 h. The apoptotic population was determined by Annexin-V-positive staining (left). Histogram of the relative apoptotic population representing an average of two independent experiments in triplicates (right). *T*-test, *P*=0.0002, *n*=6.

To assess whether the effect of KLF12 observed on cell cycle profile is translated into an effect on cell growth, IncuCyte live cell imaging was performed on A549 and H460 cells with KLF12 silencing. Measurements taken every 3 min showed a clear decrease in relative growth of cells that had knocked down KLF12 compared with scrambled control (Figure 5a). Moreover, population doublings were almost halved in A549, H460 and HOP62 cells with KLF12 knockdown relative to scrambled control (Figure 5b).

We wanted to determine whether the cell cycle role of KLF12 was linked to its role in anoikis. To this effect, we used low doses of the S-phase inhibitor aphidicolin to slow down the cell cycle in an attempt to mimic the effect of KLF12 knockdown in A549 (Supplementary Figure S5). Aphidicolin treatment had little effect on Annexin V profiling when grown on plastic. However, it was able to significantly reduce the apoptotic population of cells grown on polyHEMA compared with those treated with a dimethyl sulphoxide control (Figure 5c). Thus we suggest that KLF12 promotes anoikis by regulating the cell cycle.

### KLF12 influences anoikis resistance *in vivo*

**Figure 6:**
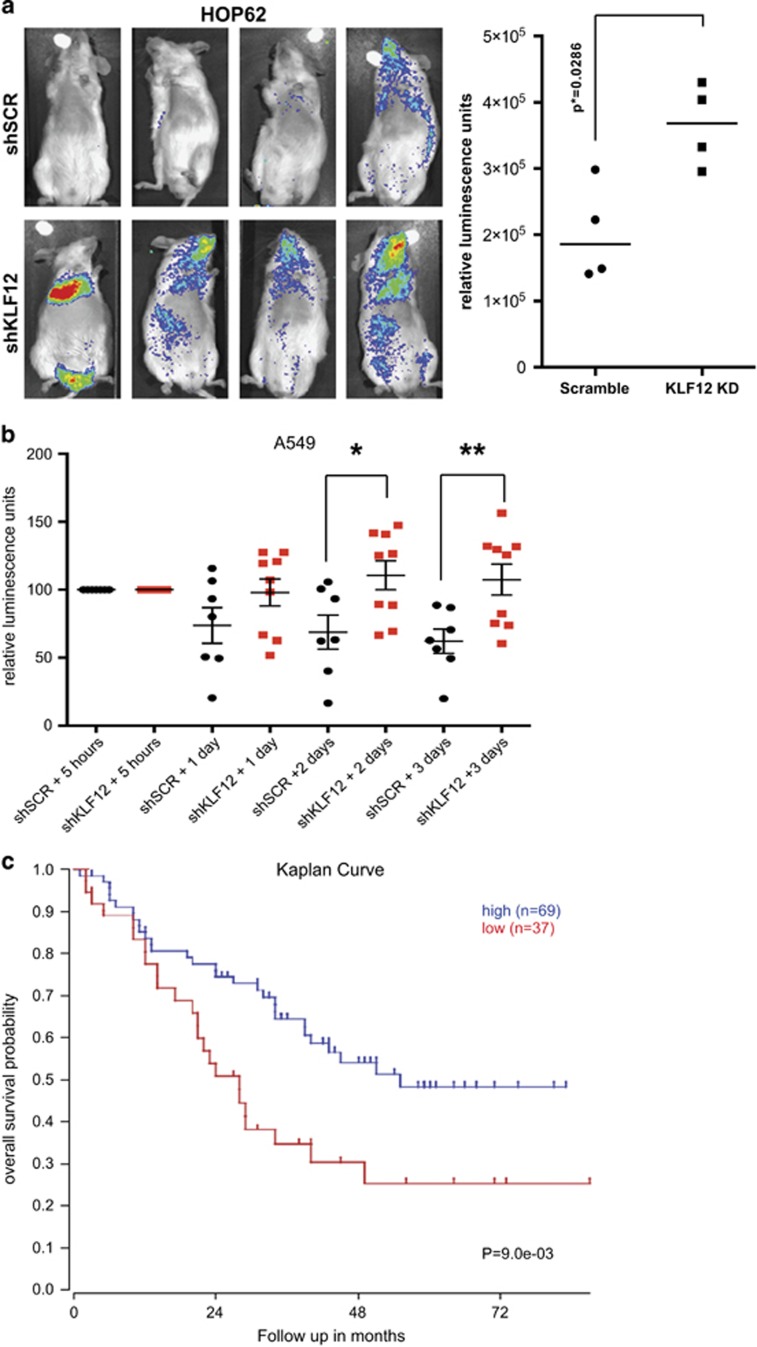
Reduced KLF12 levels result in increased lung tumour formation and decreased survival. (**a**) KLF12 knockdown results in increased HOP62 cells tumour formation. Luminescence was presented visually (left panel), and its quantification is shown in a histogram (right panel). The *P*-value was derived from Mann–Whitney test. (**b**) KLF12 knockdown results in increased A549 cells in the lung. Luminescence was quantified after tail-vein injection at set time points. Values were normalized at the 5-h time point. (**c**) The lung cancer data set from Bild *et al.*^30^ was analysed using the R2 genomics analysis and visualization platform (http://hgserver1.amc.nl/cgi-bin/r2/main.cgi).

To assess whether *KLF12* has a tumour-suppressor role *in vivo*, the gene was knocked down in HOP62, the cell line studied which expresses the highest levels of KLF12 and is the most sensitive to KLF12 knockdown *in vitro*. Luciferase-expressing HOP62 cells were infected with either control scrambled shRNA or shRNA against KLF12 and were injected into the tail vein of immunodeficient mice. Fifteen weeks after injection, the HOP62 cells that had decreased KLF12 expression could be visualized with increased signal relative to the control cells (Figure 6a).

An experimental metastasis assay was also performed with luciferase-expressing A549 cells infected with scrambled control or shRNA against KLF12. As the majority of cells are cleared soon after injection, the luciferase activity was monitored at 5 h, 1day, 2 days and 3 days postinjection. The signal of all samples were normalized at 5 h after injection, and within 2 days a statistical difference was noticed with the KLF12-knockdown cells having a higher signal in the lungs compared with the scrambled control (Figure 6b). This difference was further increased at 3 days postinjection. The scrambled control-infected cells had progressively lower signals as the days progressed, suggesting that the cells underwent anoikis. On the other hand, the cells with KLF12 knockdown seemed to either remain at a similar level to the initial luciferase signal or have a decreased signal, suggesting that whereas some cells were dying, others could survive albeit not proliferate significantly. This fits with the previous data that suggest that KLF12 knockdown can promote anoikis resistance but in parallel slows down the cell cycle. Thus KLF12 expression can affect both short-term and long-term survival in a tail-vein assay.

To assess the role KLF12 might have in a clinical setting, a publicly available microarray database that is correlated with lung cancer patient survival was analysed using a cutoff of 37 tumour samples based on KLF12 expression levels.^30^ Patients with higher KLF12 mRNA levels had increased overall survival relative to those with lower KLF12 levels (Figure 6c). Indeed, at a survival probability of 0.5, patients with higher KLF12 expression levels survived a further 24 months compared with those with lower expression levels. Thus KLF12 could act as a potential tumour suppressor in lung cancer.

## Discussion

Suppression of anoikis is an early step in the cascade leading to metastasis. The regulation of this specialized form of apoptosis is complex and still only partly understood. In the present study, we identify KLF12 as a novel candidate tumour-suppressor gene involved in detachment-induced cell death. KLF12 promotes the G1/S transition of the cell cycle and by doing so makes the cell more susceptible to undergo anoikis. Knocking down KLF12 results in increased survival of lung cancer cells growing in suspension as well as increased tumour formation in a mouse experimental metastasis model. Moreover, low KLF12 expression levels correlate with reduced survival in lung cancer patients. Although tumour suppressors have traditionally been thought of as inhibitors of the cell cycle, it has previously been demonstrated that blocking the cell cycle could confer anoikis resistance in the MCF10A model,^14^ and we confirm here that aphidicolin treatment could mimic KLF12 knockdown effect on anoikis resistance.

RNAseq analysis of A549 cells showed that a significant number of genes were differentially expressed following KLF12 knockdown. Although KLF12 had previously been described as a transcriptional repressor, our analysis showed genes that were both upregulated and downregulated. This could be due to an indirect effect whereby KLF12 represses another transcriptional repressor or to the fact that KLF12 could repress as well as transactivate its targets. Indeed, whereas KLF8 was also identified as being a transcriptional repressor by recruiting CtBP, it has been shown to transactivate certain genes as well,^31, 32, 33^ raising the possibility that KLF12 could repress as well as transactivate its targets. A chromatin immunoprecipitation-seq analysis would tell us which of those genes are direct as opposed to indirect targets of KLF12. Unfortunately, we were unable to perform those experiments owing to the unsuitability of the antibodies currently available.

KLF12 has previously been reported to have an oncogenic role in gastric and head and neck cancers,^19, 20^ although other studies suggest KLF12 might have a tumour-suppressive role. Other KLFs have been reported to be tumour suppressors or oncogenes, with their function dependent on tumour types or cancer stage.^15^ For example, KLF5 is growth promoting in non-transformed epithelial cells, while it has growth-inhibitory properties in colon tumour-derived cells.^34^ This difference of KLF5 effect was dependent on whether the cells expressed wild-type or mutant p53 owing to competition between KLF5 and p53 to bind p21 promoter.^15, 35^ Thus the discrepancy between KLF12 role as an oncogene or a tumour suppressor is likely to be context dependent.

Mutant Ras is known to have a role in anoikis resistance,^10, 36, 37, 38, 39, 40, 41, 42^ and it is worth noting that the cell lines used in these experiments, A549, H460, H23 and HOP62, all contain a mutant K-Ras gene. It is as yet unclear whether the Ras mutant status has any effect on KLF12 and its role in anoikis suppression, but this would be worth addressing in the future. Interestingly, although prostate cancer is not commonly associated with Ras mutations, there is also a correlation between KLF12 expression levels and survival of prostate cancer patients (Supplementary Figure S6).^43^ This raises the possibility that the tumour-suppressive role of KLF12 might be independent of Ras mutational status and also suggests that KLF12 might have a broader role in carcinogenesis, not limited to lung cancer.

For metastasis to occur, cancer cells need not only be resistant to anoikis but also proliferate at distant sites. As we have seen, decreased KLF12 expression leads to the former but slows down proliferation rather than promotes it. We hypothesize that KLF12 downregulation is an early step in metastasis that allows cells to adapt to anchorage-independent cell growth conditions and gives them time for further genetic or epigenetic selection to occur. The diversity in signalling and metabolism the different cell lines have acquired, such as increased P-Erk in A549, elevated p-Src and decreased OCR in H23 and higher levels of P-Akt in H460 cells, might be secondary resistance steps that occur after selection of decreased KLF12 levels.

We thus propose that anoikis resistance is a multi-step process and that loss of KLF12 expression occurs at an early stage. This allows tumour cells to adapt further to anoikis resistance in a variety of ways, so that the cells can then proliferate at distal sites.

## Materials and methods

### Cell culture, viability and proliferation assays

The expression of the luciferase enzyme in the cell lines was monitored using an IVIS Spectrum Pre-Clinical *In Vivo* Imagine System (Caliper Life Sciences, Hopkinton, MA, USA). Doxorubicin was purchased from Calbiochem (Billerica, MA, USA), Aphidicolin and polyHEMA from Sigma (St Louis, MO, USA) and were used as previously described.^44^ Viability assays were performed as previously described.^45^ Cell growth/confluence was measured in real time on an Incucyte FLR (Essen Instruments, Ann Arbor, MI, USA). Proliferation in culture was assayed as previously described.^46^

### Cloning, reverse transcriptase Q-PCR and antibodies

KLF12 was PCR amplified from a human cDNA library introducing an N-terminal FLAG tag and sequenced. Oligos are described in Supplementary Table S1a. The luciferase gene was cloned into BamH1/SalI cloning sites of a pBabe-Blastocidin retroviral expression vector. Primer details for reverse transcriptase Q-PCR and antibody details are provided in Supplementary Tables S1d and e, respectively.

### siRNA transfection and shRNA production and infection

Cells were transfected with siRNAs by reverse transfection using Dharmafect 1 as per the manufacturer's methods (Dharmacon, Lafayette, CO, USA). Retroviral and lentiviral production and infection were performed as previously described.^47^ shRNA and siRNA details are in Supplementary Tables S1b and c, respectively.

### Flow cytometry

For cell cycle profiling, adherent and suspension cells were collected, fixed with 70% ethanol and stained with propidium iodide. For BrdU treatment, cells were treated with 10 μM for 1 h after which the cells were harvested and fixed as described above. For BrdU pulse-chase assays, cells were pulsed with 10 μM BrdU for 15 min after which they were washed with phosphate-buffered saline and grown in full serum for the indicated times prior to harvesting, as described above. For apoptosis analysis, floating and adherent cells were harvested, resuspended in Binding Buffer (BD Biosciences, Franklin Lakes, NJ, USA), stained with Annexin V–fluorescein isothiocyanate antibody and propidium iodide and analysed by flow cytometry.

### Metabolic assays

O_2_ consumption were measured using the Seahorse XFe Extracellular Flux Analyzer (North Billerica, MA, USA) according to the manufacturer's standard protocol. For normalization, cells were seeded in parallel in a 96-well plate, fixed in 70% ethanol and stained with 4,6-diamidino-2-phenylindole (DAPI; 1 μg/ml DAPI in phosphate-buffered saline) for 1 h. The cell count was measured by the Acumen eX3 Analyzer (TTP Labtech, Hertfordshire, UK) by determining the total area of DAPI staining and dividing it by the area of a single nucleus.

### Gene expression analysis

The NimbleGen microarray was performed in triplicates following the manufacturer's protocol (Roche, Basel, Switzerland). Four micrograms of Cy3-labelled double-stranded cDNA was hybridized to a NimbleGen Gene Expression array. The array data were normalized by Robust Multi-array Average. Statistical significance was assessed by one-way analysis of variance with a false-discovery rate of 0.05. The analysis was carried out using Limma from Bioconductor. We selected differential probes using a 0.05 false-discovery rate and a fold difference of at least twofold in each of the different cell lines.

### RNASeq quantitation

Illumina (San Diego, CA, USA) 72 base pair single-ended reads were aligned to UCSC human genome reference hg19 (40e6 reads/sample)^48^ using the Tophat2 algorithm.^49^ KnownGene annotations from UCSC provided transcript annotations for the reference genome. Differential expression levels between cell line and control samples were quantified for the known genes using Cuffdiff.^50^ Gene lists for each sample–control pair comprised those that were selected from the Cuffdiff results, which were differentially expressed at a *P*-value<0.001. Venn logic was applied to the lists to obtain those genesets specific to each sample–control pair.

### Experimental metastasis assays

Hop62 and A549 cells expressing stable levels of luciferase were suspended in phosphate-buffered saline to a concentration of 10^6^ cells/0.2 ml. A total of 0.2 ml of these solutions was injected into the tail vein of different groups of Nod/Scid Gamma mice. Bioluminescence emission of the cells injected was monitored at different time points using an IVIS Spectrum Pre-Clinical *In Vivo* Imagine System according to the manufacturer's instructions.
