# Supplementary figures and images for: Tumour-suppression function of KLF12 through regulation of anoikis
Source: Oncogene. 2015 Oct 12;35(25):3324–34. doi: 10.1038/onc.2015.394 (PMC4929484; doi:10.1038/onc.2015.394)

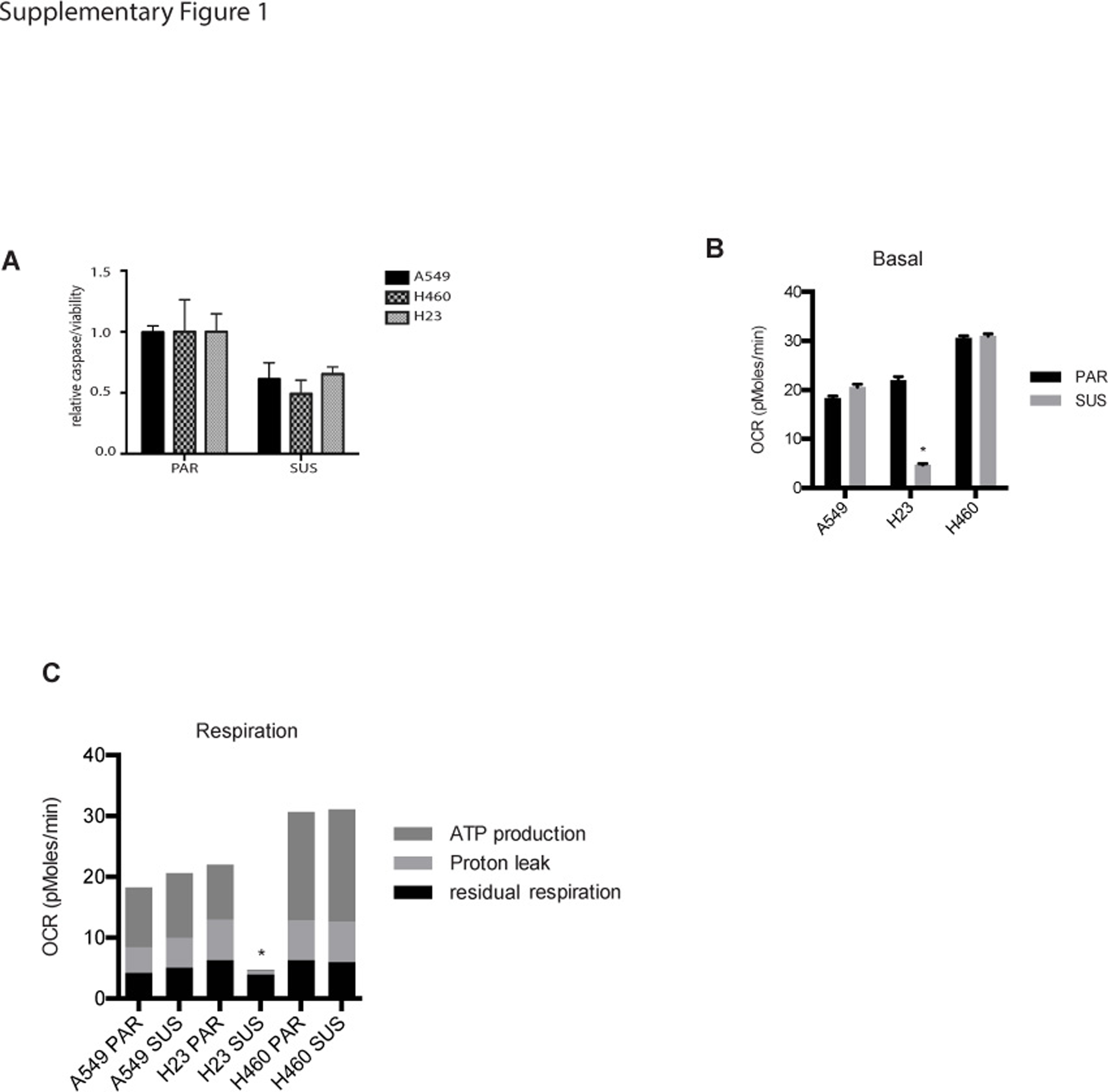

Supplement: Supplementary Figure 1 [file onc2015394x1.tif]

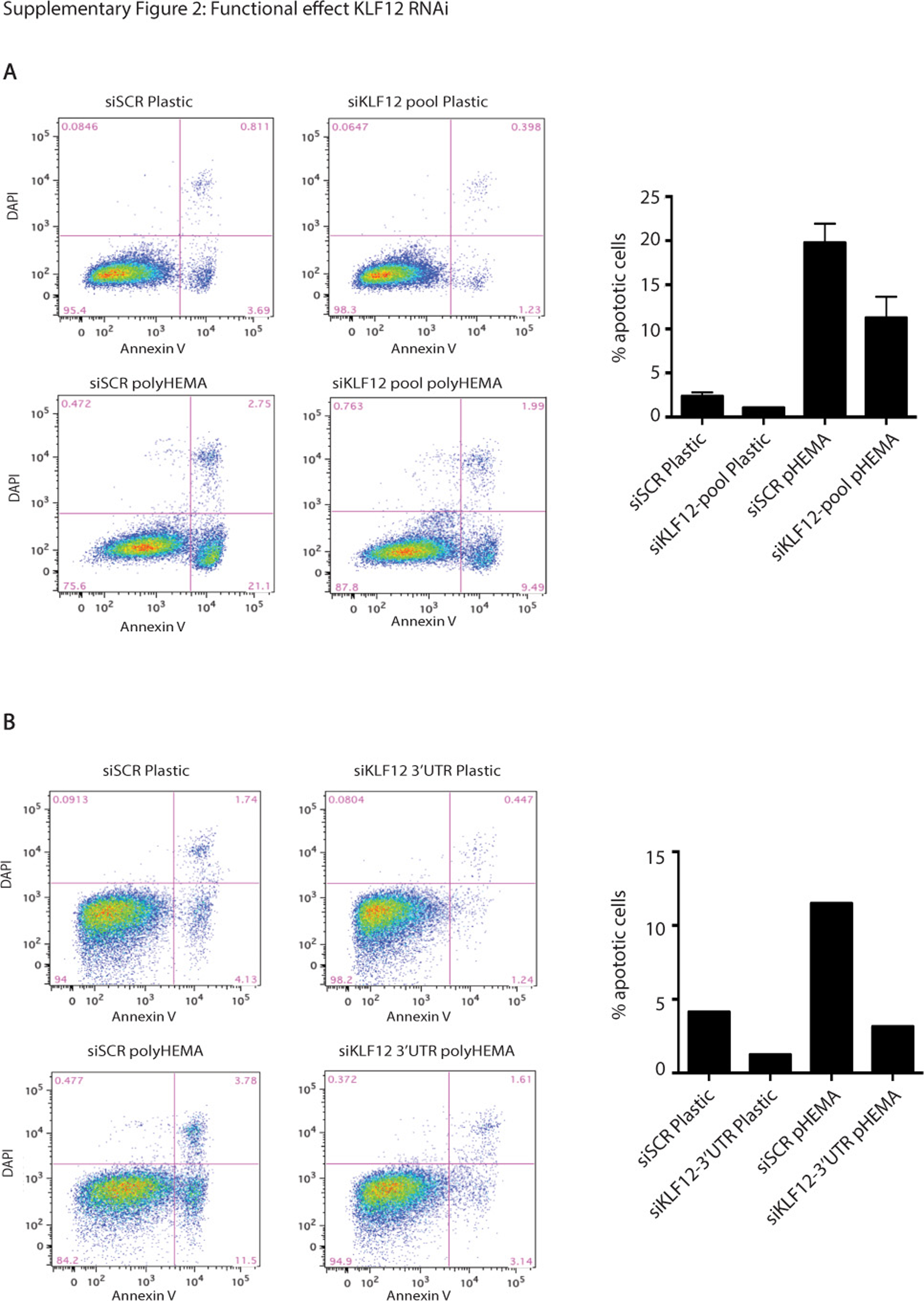

Supplement: Supplementary Figure 2 [file onc2015394x2.tif]

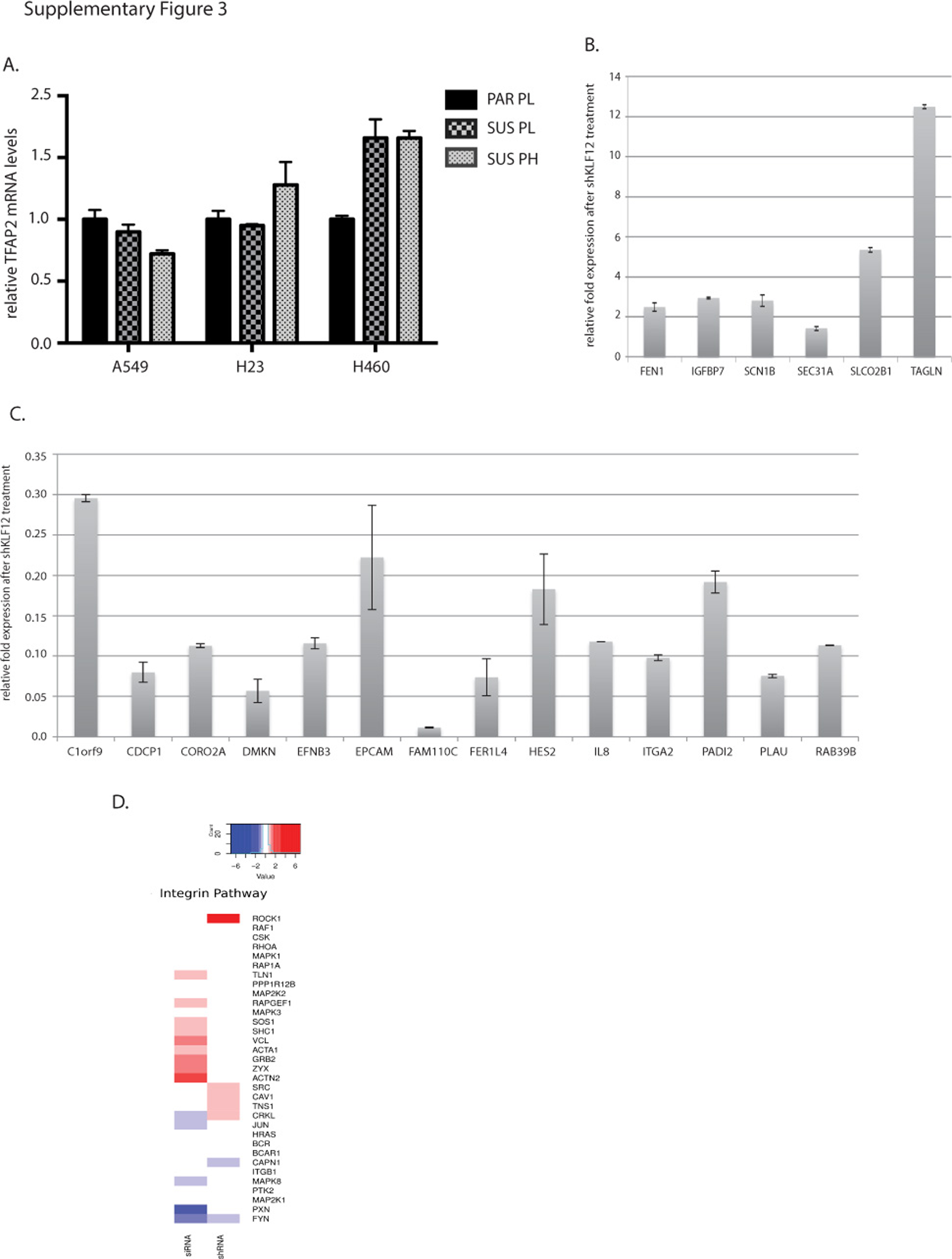

Supplement: Supplementary Figure 3 [file onc2015394x3.tif]

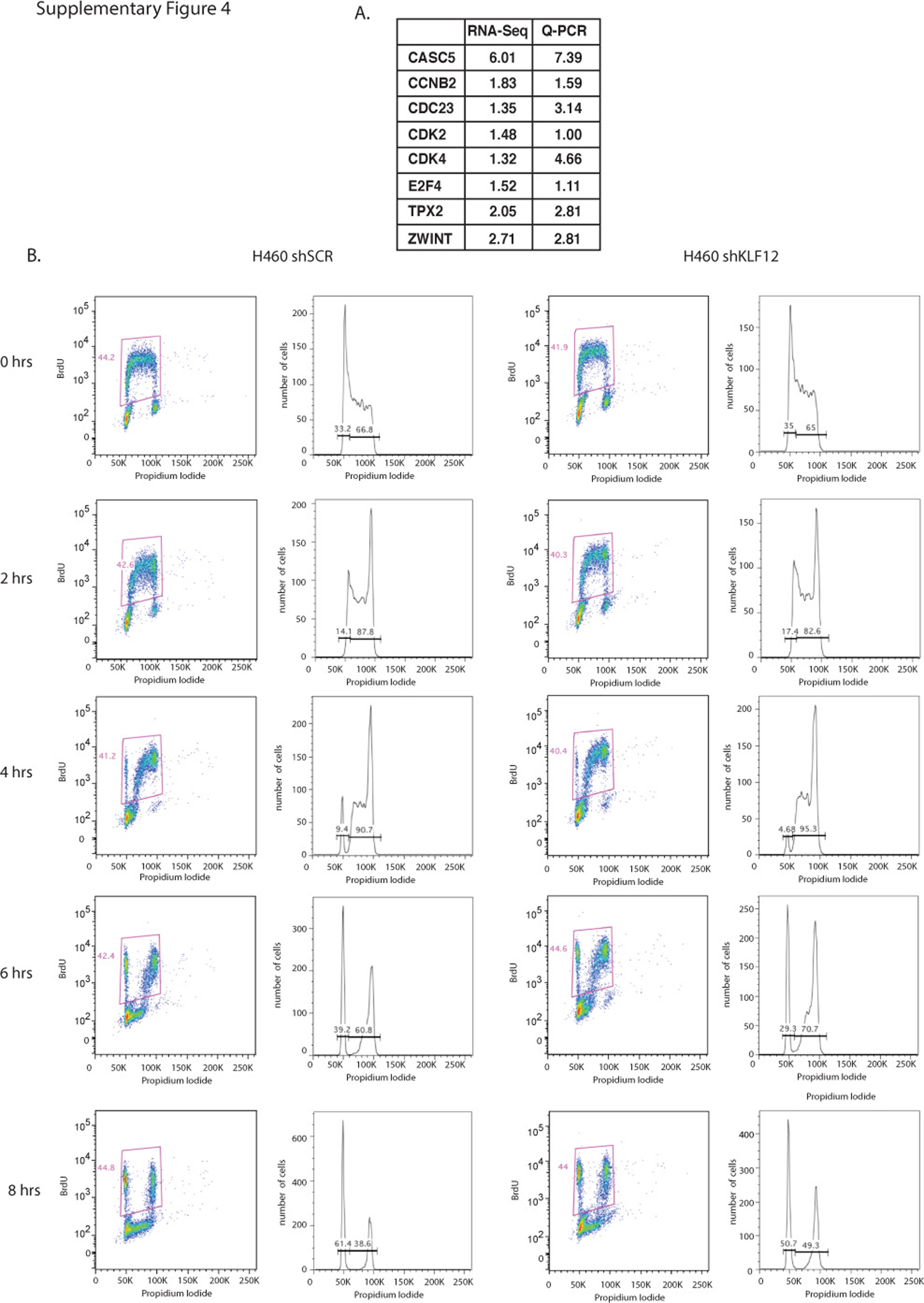

Supplement: Supplementary Figure 4 [file onc2015394x4.tif]

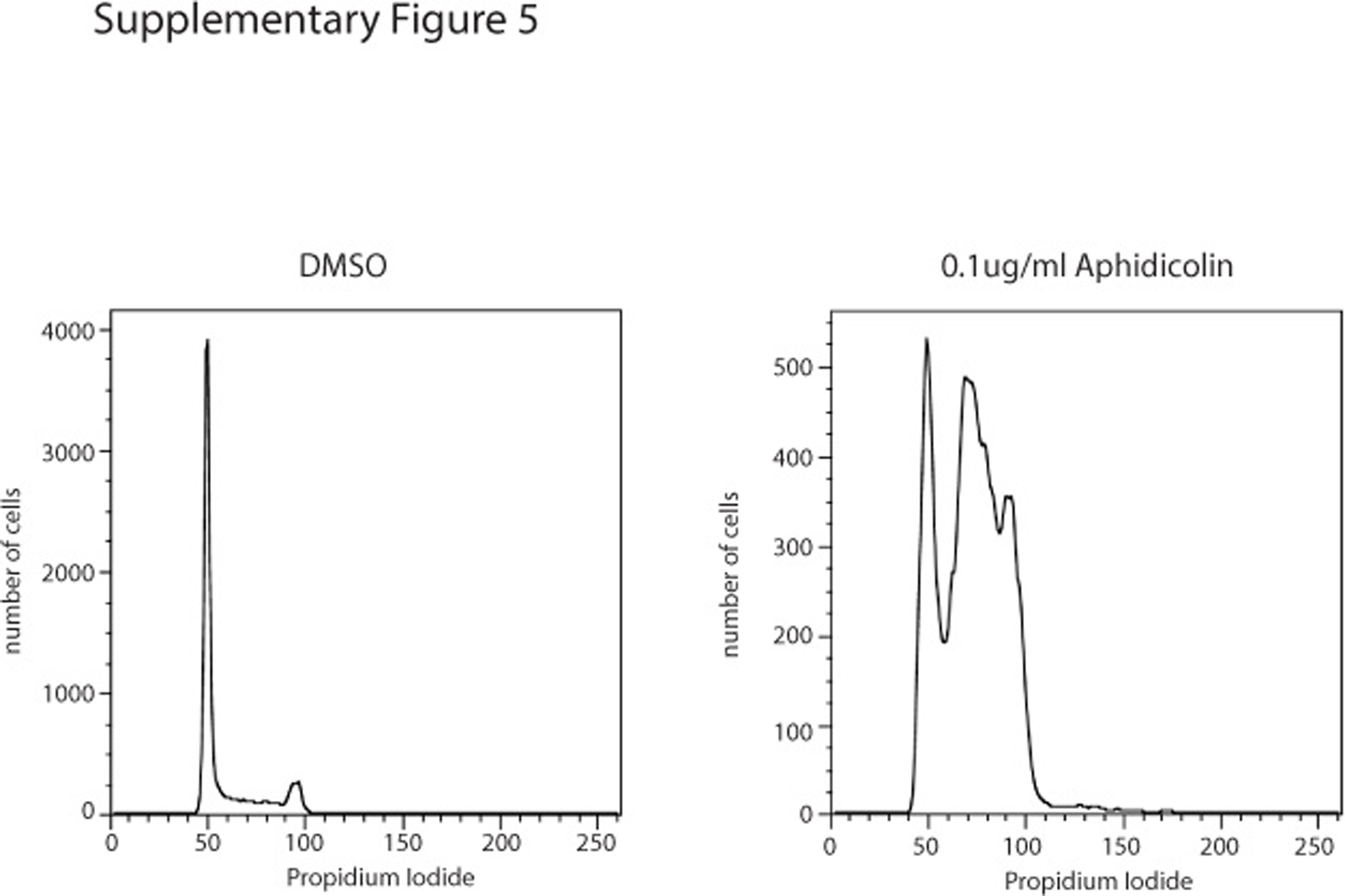

Supplement: Supplementary Figure 5 [file onc2015394x5.tif]

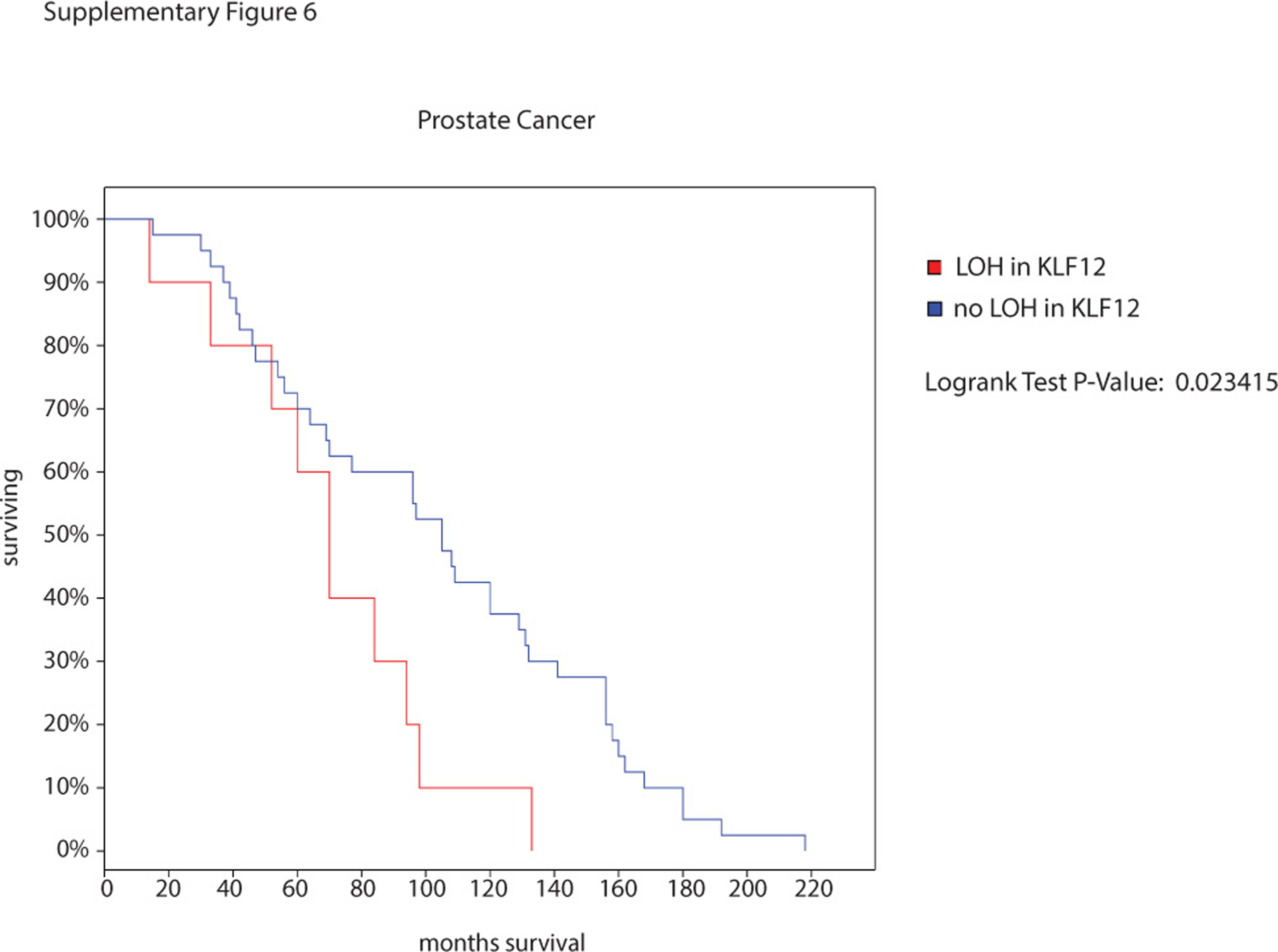

Supplement: Supplementary Figure 6 [file onc2015394x6.tif]
