# Supplementary material for: Tumour-suppression function of KLF12 through regulation of anoikis
Source: Oncogene. 2015 Oct 12;35(25):3324–34. doi: 10.1038/onc.2015.394 (PMC4929484; doi:10.1038/onc.2015.394)
Supplement: Supplementary Tables [file onc2015394x7.doc]

**SUPPLEMENTARY TABLES**

| Oligo | Sequence | Use |
| --- | --- | --- |
| FOR EcoRI flag KLF12 | catgaattcccaccatggattacaaggatgacgatgacaagaatatccatatgaagagaaaaacaataaag | Cloning |
| REV EcoRI KLF12 primer | catgaattctcacaccaacatatgcctcc | Cloning |
| FOR KLF12-539 | tgagtcatgttcaccgcatc | Sequencing |
| REV KLF12-957 | tgttgcatccctcaaaatca | Sequencing |
| FOR KLF12-915 | gtctgaatccccagactcca | Sequencing |
| REV KLF12-366 | cagttggggatgaggctaga | Sequencing |

**Supplementary Table 1a. Oligonucleotide sequences**

| shRNA | TRC catalogue no. |
| --- | --- |
| Human shKLF12 | TRCN0000015804 |
| Human shGAPDH | TRCN0000025828 |

**Supplementary Table 1b. shRNA sequences**

| siRNA | Company | catalogue no. |
| --- | --- | --- |
| siscrambled | Dharmacon | siCONTROL D-0001210-02 |
| siKLF12- 1 | Dharmacon | siGENOME D-013353-01 |
| siKLF12- 1 | Dharmacon | siGENOME D-013353-02 |
| siKLF12- 1 | Dharmacon | siGENOME D-013353-03 |
| siKLF12- 3'UTR | Sigma | SASI_Hs02_00343275 |

**Supplementary Table 1c. siRNA sequences**

| Transcript | Company | qRT-PCR catalogue no. |
| --- | --- | --- |
| KLF12 | Applied Biosystems | Hs00971557_m1 |
| GAPDH | Applied Biosystems | 4333764 |
| TFAP2A | Qiagen | QT00085225 |
| GAPDH | Qiagen | QT01192646 |

**Supplementary Table 1d. qRT-PCR primers**

| Protein | Company | Catalogue no. |
| --- | --- | --- |
| P42 MAP Kinase | Cell Signalling | 9108 |
| Phospho-p44/42 MAP Kinase (T202/Y204) | Cell Signalling | 9488 |
| Total Src | Cell Signalling | 2110 |
| Phospho-Src Family (Y416) | Cell Signalling | 2101 |
| Phospho-FAK (Y397) | Invitrogen | 36-7900 |
| Total FAK | Abcam | 105917 |
| Bim | BD Pharmingen | 559685 |
| Phospho-Akt/PKB (S473) | Biosource | 44-621G |
| Akt/PKB | Cell Signalling | 9272 |
| β-actin | Sigma | A1978 |
| KLF12 | Proteintech Europe | 13156-1-AP |

**Supplementary Table 1e. Antibodies**

**SUPPLEMENTARY FIGURE LEGENDS**

**Supplementary Figure 1: Characterisation of PAR and SUS cells**

1. A549, H460 and H23 suspension-derived sublines have lower levels of apoptosis as determined by caspase activity relative to their parental cell lines when grown on polyHEMA. A t-test showed the P value comparing the PAR and SUS samples to be 0.136, 0.11 and 0.08 for A549, H460 and H23, respectively.
2. A549 PAR, A549 SUS, H23 PAR, H23 SUS, H460 PAR and H460 SUS cells were seeded on plastic at high densities and the following day were subjected to a Seahorse xF Analysis. Basal oxygen consumption rates (OCR) of A549 PAR, A549 SUS, H23 PAR, H23 SUS, H460 PAR and H460 SUS cells. Values were normalized to 10,000 cells, counted via parallel nuclei staining.
3. Basal OCRs of A549 PAR, A549 SUS, H23 PAR, H23 SUS, H460 PAR and H460 SUS were measured prior to 1 μg/ml oligomycin, 1 μM antimycin A, 1 μM rotenone treatment and a subsequent measurement of OCR. The illustrated values were generated as the difference between the subsequent measurements:

OCR_ATP production_ = OCR_basal_ – OCR_oligomycin_

OCR_proton leak_ = OCR_oligomycin_ – OCR_antimycin A/rotenone_

OCR_non-mitochondrial respiration_ = OCR_basal_ – OCR_antimycin A/rotenone_

Data are presented as mean + s.e.m.; n ≥ 6. *: P < 0.0001 compared with the PAR histogram bar.

**Supplementary Figure 2: siRNA against KLF12 reduces apoptotic population**

1. A549 PAR cells infected with pooled siRNA against KLF12 or scramble control were grown in polyHEMA or plastic and the Annexin-positive apoptotic population was quantified by FACS analysis (left). Average of annexin-positive apoptotic population of six independent experiments (right).
2. A549 PAR cells infected with a siRNA against the 3’-UTR of KLF12 or scramble control were grown in polyHEMA or plastic and the Annexin-positive apoptotic population was quantified by FACS analysis (left). Summary of annexin-positive apoptotic population (right).

**Supplementary Figure 3: KLF12 regulates the cell cycle**

1. TFAP2 mRNA levels are not significantly different in PAR versus SUS cells across all three cell lines as determined by Q-PCR.
2. Validation by Q-PCR of genes up-regulated in A549 after shRNA against KLF12 in RNAseq.
3. Validation by Q-PCR of genes down-regulated in A549 after shRNA against KLF12 in RNAseq.
4. Heatmap of integrin signature from genes differentially expressed following KLF12 knockdown by shRNA and siRNA. The integrin signature was taken from the BIOCARTA gene list and is statistically significant for the siRNA sample (P=0.00065) but not for the shRNA sample.

**Supplementary Figure 4: shRNA against KLF12 delays S phase exit in H460**

1. Fold difference of expression for cell cycle genes differentially expressed following KLF12 knockdown by both siRNA and shRNA. The table shows the values for the RNASeq and validated Q-PCR following siKLF12 relative to scramble.
2. BrdU was pulsed for 15 minutes in H460 cells stably expressing shRNA against KLF12 or scramble control, then chased with BrdU-free medium and cells were fixed at indicated time points. For each time point and sample, BrdU-positive cells cycling through S phase are gated (left), and the DNA content of those cells are depicted (right). The percentage of cells in early S phase and mid-late S phase is shown.

**Supplementary Figure 5: Low doses of Aphidicolin results in delayed S phase exit**

1. A549 PAR cells were treated with 0.1 μg/ml Aphidicolin or DMSO on plastic for 48 hours prior to cell cycle profiling.

**Supplementary Figure 6: Reduced KLF12 expression correlates with reduced survival in prostate patients**

1. The prostrate cancer database from Grasso et al. was analysed using <http://www.cbioportal.org/public-portal/>. The log-rank P value is 0.023415.
